# Quality of Red Blood Cells Isolated from Umbilical Cord Blood Stored at Room Temperature

**DOI:** 10.1155/2012/102809

**Published:** 2011-12-14

**Authors:** Mariia Zhurova, John Akabutu, Jason Acker

**Affiliations:** ^1^Department of Laboratory Medicine and Pathology, University of Alberta, 8249-114 Street, Edmonton, AB, Canada T6G 2R8; ^2^Alberta Cord Blood Bank, Suite 408 College Plaza, 8215-112 Street, Edmonton, AB, Canada T6G 2C8; ^3^Research and Development, Canadian Blood Services, 8249-114 Street, Edmonton, AB, Canada T6G 2R8

## Abstract

Red blood cells (RBCs) from cord blood contain fetal hemoglobin that is predominant in newborns and, therefore, may be more appropriate for neonatal transfusions than currently transfused adult RBCs. Post-collection, cord blood can be stored at room temperature for several days before it is processed for stem cells isolation, with little known about how these conditions affect currently discarded RBCs. The present study examined the effect of the duration cord blood spent at room temperature and other cord blood characteristics on cord RBC quality. RBCs were tested immediately after their isolation from cord blood using a broad panel of quality assays. No significant decrease in cord RBC quality was observed during the first 65 hours of storage at room temperature. The ratio of cord blood to anticoagulant was associated with RBC quality and needs to be optimized in future. This knowledge will assist in future development of cord RBC transfusion product.

## 1. Introduction

Fetal and neonatal anemias are among the most serious complications of pregnancy and postnatal development. The causes of fetal anemia include immune haemolytic disease [1], defects in hemoglobin structure and synthesis, fetomaternal or twin-to-twin hemorrhages, and parvovirus B19 infections [2]. Neonatal anemia, on the other hand, can either result from fetal anemia or develop after birth as a result of hemorrhage due to obstetric accidents, frequent drawing of blood for laboratory testing, or impaired red blood cell (RBC) production by bone marrow [2]. The most commonly used treatments for fetal and neonatal anemia are transfusions of red blood cells (RBCs), either intrauterine [1] or intravenous [3–6], to help replace the lost RBCs of the fetus or neonate.

RBCs used in intrauterine and neonatal (intravenous) transfusions are derived from adult donors [1, 3–7]. Adult RBCs are different from those present in the blood of a fetus or neonate [2, 8–12]. Neonatal RBCs obtained from umbilical cord blood (cord RBCs) are usually discarded during isolation of stem cells from cord blood [13–15]. This waste product may offer a superior alternative for intrauterine and neonatal transfusions [16, 17].

Cord RBCs are unique cells that differ from adult RBCs in membrane composition and biophysical properties [2, 9], hemoglobin (Hb) structure [2, 8–12, 18], metabolism, and enzymatic profile [8, 10]. One of the most important physiological differences is the high concentration of fetal hemoglobin (HbF) in cord RBCs. This is practically absent in adult RBCs (normal adult HbF is <1%) [18]. HbF has a higher affinity for oxygen compared to adult hemoglobin (HbA). This allows HbF to bind oxygen more easily, with a left shift of the oxygen dissociation curve and the release of less oxygen to the tissues [18].

There are many problems associated with adult RBC transfusions to fetuses and neonates that can be resolved by replacing adult RBCs with cord RBCs. Due to the high concentration of HbF, fetal blood has higher affinity for oxygen than the mother's blood, which facilitates the uptake of oxygen from the placenta by the fetus [11]. The practice of administering adult RBC transfusions to premature infants has been associated with the increased incidence of retrolental fibroplasia—the abnormal growth of blood vessels in the retina that may lead to blindness [19–21]. Another possible complication of adult RBC transfusions to neonates is bronchopulmonary dysplasia, a chronic inflammatory lung disease that can lead to respiratory dysfunction [22]. Several researchers have demonstrated a direct correlation between the incidence of bronchopulmonary dysplasia and adult blood transfusions [23–25].

The practice of transfusions of RBCs derived from umbilical cord blood to treat neonatal anemia has recently gained a lot of interest [26–35]. A number of studies have demonstrated that transfusions of autologous cord RBCs are both safe and effective in the treatment of anemic neonates [26–32]. Some, however, have expressed concerns with cord blood transfusions, including the potential high risk of bacterial contamination, low hypothermic storage stability, and small volume of umbilical cord blood collections [28, 33]. At the same time, these can be overcome through use of novel or superior long-term storage techniques for cord RBCs.

Adult RBCs can be successfully stored at 1–6°C in an anticoagulant/preservative solution (e.g., citrate-phosphate-dextrose/saline-adenine-glucose-mannitol (CPD/SAGM)) for 42 days [36]. In contrast, cord RBCs deteriorate much faster under the same conditions and cannot be stored for more than 14 days without significant decrease in quality [28, 37]. Cryopreservation and subsequent storage at ultra-low temperatures may preserve cord RBCs and maintain a high quality of cord RBCs for use in intrauterine and neonatal transfusions. Despite many studies having documented the successful cryopreservation of adult RBCs, no protocol for cryopreservation of cord RBCs has been developed.

Since cord RBCs are currently treated as a waste product after processing of collected cord blood for stem cell extraction, there is no incentive to monitor or preserve their quality. After cord blood is collected, it can be stored ideally at room temperature for up to 48 hours before being processed for stem cell extraction [38]. Longer pre-processing room temperature storage for stem cell extraction is permissible when there is strict monitoring of cell viability, CD 34+ cell content and viability, and colony-forming assay potential. Presently, the effects of pre-processing storage on the quality of cord RBCs is unknown.

A number of conventional methods exist for assessing RBC quality. RBC hemolysis is used as an indicator of RBC membrane damage that results in the release of free Hb into extracellular space. A decrease in adenosine triphosphate (ATP), the energy substrate of RBCs, has been observed as one of the markers of RBC aging during hypothermic storage [39]. The concentration of 2,3-diphosphoglycerate (2,3-DPG) in RBCs is another important quality parameter, since 2,3-DPG regulates oxygen exchange between Hb and tissues by mediating the binding of oxygen to Hb, as well as the release of oxygen into tissues [8, 40]. Finally, the concentration of methemoglobin (metHb) acts as an indicator of RBC oxidative injury.

In addition, novel predictors of RBC *in vitro *quality, such as RBC deformability, microvesiculation, and extracellular expression of phosphatidylserine (PS) and CD47, have been recently incorporated into RBC quality assessments [41, 42]. The deformability of RBCs enables their passage through small blood capillaries and is therefore a critical property for maintaining normal blood flow [43]. Microvesiculation is the process of generating microparticles—phospholipid vesicles 0.04–1.5 *μ*m in diameter—by eukaryotic cells as a result of different types of cell stimulation [44]. Notably, microvesiculation has been shown to increase during hypothermic storage of adult RBCs [45]. Glycophorin A (CD235a) is a glycoprotein abundantly present on the RBC membrane [46], and its expression on microparticles is used as a marker of RBC origin [44]. PS is a membrane phospholipid that is normally found within the inner leaflet of the plasma membrane, but during apoptosis, it is translocated to the outer leaflet. Annexin V is a phospholipid-binding protein with a high affinity for PS and is used to determine the percentage of cells within a population that are actively undergoing apoptosis. It has been shown that PS is exposed on the surface of RBCs during hypothermic storage [47]. CD47 is an erythrocyte surface antigen that has been shown to be a “marker of self.” RBCs lacking this antigen on their surface are rapidly cleared from the circulation by macrophages in the spleen [48]. It has been demonstrated that the expression of CD47 on RBCs decreases during storage and may be an important determinant of posttransfusion *in vivo* survival [49].

If cord RBCs are to be cryopreserved for clinical use, it is critical to ensure that a high quality of cord RBC product remains, following isolation from cord blood. The objective of the present study was to examine if and how the quality of cord RBCs is affected by the duration of cord blood storage at room temperature and other cord blood characteristics.

## 2. Methods

### 2.1. Cord RBC Collection

Cord RBCs were obtained from the Alberta Cord Blood Bank, as a waste product, after stem cell isolation from umbilical cord blood. Women with healthy, full-term pregnancies who met the Alberta Cord Blood Bank criteria for inclusion and gave informed consent were eligible to donate cord blood. Cord blood collections were performed by a trained physician or midwife attending the delivery, from either the undelivered or delivered placenta. The umbilical vein was punctured, and cord blood was collected by gravity into a blood collection bag (Fenwal, Inc., Lake Zurich, IL, USA) containing 35 mL of citrate phosphate dextrose (CPD) anticoagulant. After collection, whole umbilical cord blood was stored at room temperature and processed at the Alberta Cord Blood Bank facility according to a previously described double collection procedure [50]. Ethics approval for the study was obtained from the University of Alberta Health Research Ethics Board (Biomedical Panel).

### 2.2. Cord Blood Characteristics

Upon receipt of a packed cord RBC unit, a number of cord blood characteristics were documented from the Alberta Cord Blood Bank Collection Form. The time between cord blood collection and the start of processing that cord blood spent at room temperature was calculated (age of cord blood). The baby's gender and weight, the cord blood collection method (*in utero* or delivered placenta), and cord blood hematocrit were recorded. The ratio of cord blood volume to anticoagulant volume (cord blood  : CPD) was calculated by dividing the reported volume of cord blood in mL (w/o anticoagulant) by 35 mL (volume of CPD in blood bag). Additionally, the percentage of HbF in cord blood was determined using the standard Kleihauer-Betke kit (Sure Tech Diagnostic, Associates, Inc., St. Louis, MO, USA).

### 2.3. Assessment of Cord RBC Quality

#### 2.3.1. Standard Indicators of RBC Quality

Cord RBCs were tested immediately after their isolation from cord blood for conventional indicators of RBC quality. RBC hemolysis was determined by spectrophotometric measurement of total and supernatant cyanmethemoglobin according to Drabkin's method [51]. Controls for total Hb were prepared from Stanbio Tri-Level Hemoglobin controls (Stanbio Laboratory, Boerne, TX, USA). Hematocrit of RBC sample was measured using the microhematocrit centrifuge (Hettich, Tuttlingen, Germany) as the ratio of the volume occupied by packed RBCs to the volume of a whole RBC sample [46]. ATP concentration in RBCs was determined using the commercial ATP Hexokinase FS kit (DiaSys Diagnostic Systems GmbH, Holzheim, Germany). A blank sample was prepared with distilled water, and a control sample was prepared using an ATP standard (DiaSys Diagnostic Systems GmbH, Holzheim, Germany). ATP concentration was subsequently converted from *μ*mol/dL to *μ*mol/g Hb using the following formula:

(1) $$\begin{array}{c} \text{C}\left(\mu \text{mol}/\text{gHb}\right)\text{=C}\left(\mu \text{mol}/\text{dL}\right)\times \frac{10}{\text{Hb}\left(\text{g}/\text{L}\right)} \end{array}$$

2,3-DPG concentration in RBCs was determined using the commercially available 2,3-DPG assay kit (Roche Diagnostics GmbH, Mannheim, Germany). Concentration of metHb was measured spectrophotometrically on the SPECTRA max PLUS 384 microplate spectrophotometer (Molecular Devices Corporation, Sunnyvale, CA, USA). RBCs were diluted 1 : 500 in distilled water, and the concentration of each type of Hb (oxyhemoglobin, metHb, carboxyhemoglobin) was determined by measuring the absorption of lysed RBCs at four different wavelengths (560 nm, 576 nm, 630 nm, and 700 nm), since each type of Hb has a unique absorption peak. Concentrations of different types of Hb were calculated as the amount of heme monomer in mol/L using the following formulas [52]:

(2) $$\begin{array}{cc} C_{\text{met}} & =501\times [\left(-0.361416\times \left(A_{560}-A_{700}\right)\right)\\ & \;\;\;\;+\left(0.174064\times \left(A_{576}-A_{700}\right)\right)\\ & \;\;\;\;+\left(2.68255\times \left(A_{630}-A_{700}\right)\right)]\times 10^{-4},\\ C_{\text{oxy}} & =\;501\times [\left(-0.741711\times \left(A_{560}-A_{700}\right)\right)\\ & \;\;\;\;+\left(1.01587\times \left(A_{576}-A_{700}\right)\right)\\ & \;\;\;\;-\left(0.279425\times \left(A_{630}-A_{700}\right)\right)]\times 10^{-4},\\ C_{\text{deoxy}} & =501\times [\left(1.35699\times \left(A_{560}-A_{700}\right)\right)\\ & \;\;\;\;-\left(0.739456\times \left(A_{576}-A_{700}\right)\right)\\ & \;\;\;\;-\left(0.671847\;\times \;\left(A_{630}-A_{700}\right)\right)]\times 10^{-4}, \end{array}\;$$

where *C* = concentration of each type of Hb (mol/L), and *A* = absorbance at each wavelength. Lastly, the percentage of metHb relative to total Hb was subsequently calculated.

#### 2.3.2. Novel Indicators of RBC Quality

RBC deformability was analyzed via ektacytometry using the laser-assisted optical rotational cell analyzer (LORCA, Mechatronics, Zwaag, The Netherlands). In ektacytometry, the RBC suspension is subjected to different levels of shear stress during rotation at different speeds, which causes the RBCs to elongate to different extents. A laser beam, shone through the RBC suspension, is refracted by RBCs, and the shape of the diffraction pattern is used to determine EI_max_, the maximum theoretical elongation index, and K_EI_, the shear stress required to achieve half of the EI_max_ [43]. A high EI max suggests that RBCs are highly deformable, whereas a high KEI means that RBCs are very rigid and, hence, more force needs to be applied for RBCs to elongate. For ektacytometry experiments, RBCs were diluted 1 : 100 in polyvinylpyrrolidone (PVP, Mechatronics, Zwaag, The Netherlands). All measurements were performed at 37°C. Deformability data was analyzed using Eadie-Hofstee linearization as previously described by Stadnick et al. [53].

Flow cytometry was used to assess RBC microvesiculation, as well as expression of PS and CD47 by both RBCs and RBC microparticles. To prepare RBC samples, RBC concentrates were diluted 1 : 5650 in Annexin V binding buffer (prepared in-house and contained 140 mM sodium chloride, 2.5 mM calcium chloride, 10 mM 4-(2-hydroxyethyl)-1-piperazineethanesulfonic acid (HEPES)) in two steps. To eliminate any small particles originally present in the buffer, the buffer was sterile filtered through 0.2 *μ*m Supor Membrane VacuCap 60 Bottle-top filters (Pall Life Sciences, Ann Arbor, MI, USA) before being used for any cell dilutions. Nine hundred eighty-five microliters of RBC suspension was then labelled with 5 *μ*L each of FITC-conjugated anti-glycophorin A (Invitrogen Corporation, Camarillo, CA, USA), APC-conjugated Annexin V (BD Pharmingen, Franklin Lakes, NJ, USA), and PE-conjugated anti-CD47 (BD Pharmingen, Franklin Lakes, NJ, USA) and incubated for at least 15 minutes in the dark at room temperature.

A number of controls were used in this assay. Unstained RBCs served as a negative control. To determine the degree of nonspecific binding of antibodies, isotype controls were prepared by labeling 990 *μ*L of RBCs with 5 *μ*L each of PE-conjugated mouse IgG1, k (BD Pharmingen, Franklin Lakes, NJ, USA) and FITC-conjugated mouse IgG1, k (Invitrogen Corporation, Camarillo, CA,USA). The positive control for RBC microparticles and PS externalization was prepared by treating fresh RBCs with N-ethylmaleimide (NEM) (Sigma-Aldrich, St. Louis, MO, USA), as previously described by Stewart et al. [54], and subsequently labeling NEM-treated RBCs with fluorescent antibodies in the same way as a test sample. Annexin V binding buffer was run alone to check for the purity of the buffer and absence of microparticle artifacts, and the Annexin V binding buffer with added fluorescent antibodies was run as a blank control.

Prepared samples were then analyzed using an FACSCalibur flow cytometer (BD Biosciences, San Jose, CA, USA) with a low flow rate. Uniform Polystyrene Microspheres 1.01 *μ*m in diameter (Bangs Laboratories, Inc., Fishers, IN, USA) were used as a size reference to set a gate around the desired population of microparticles, and only microparticles less than 1.01 *μ*m in diameter were included in the analysis. Only microparticles positive for Glycophorin A were considered to be of RBC origin and, therefore, further quantified and analyzed for PS and CD47 expression. Data analysis was performed using CellQuest Pro software, Version 6.0 (BD Biosciences, San Jose, CA, USA). After analysis, flow cytometry output data was used to calculate the percentage of microparticle events in each RBC sample, the percentage of RBCs and RBC microparticles expressing PS and CD47, and the mean fluorescence intensity (MFI) of PS and CD47 on RBCs and RBC microparticles.

### 2.4. Statistical Analysis

Three types of statistical analysis were used in the present study: correlation, regression, and Student's *t*-test. Statistical analysis was conducted using statistical analysis system (SAS) software, version 9.1 (SAS institute Inc., Cary, NC, USA). To investigate the strength of the relationship between cord blood characteristics and cord RBC quality parameters, correlation analysis was performed. Spearman rank coefficients were calculated for the baby's gender and cord blood collection site, which are discrete variables, while Pearson's correlation coefficients were calculated for other cord blood characteristics. The use of regression analysis permitted the effects of multiple cord blood characteristics on RBC quality measures to be examined in a model simultaneously. In regression analysis, the backward selection approach was used to select the variable(s) for the final statistical model. First, all of the characteristic variables were entered into the model. Then, variables possessing the highest *P* values (indicating no effect) were removed one by one until all of the remaining variables comprising the model had a significance of a *P* value <0.1. Student's *t*-test was used for comparison of cord blood characteristics and cord RBC quality parameters between groups of cord blood units based on baby's gender and a method of cord blood collection. The significance level was set to 0.05. 

## 3. Results

**Figure 1:**
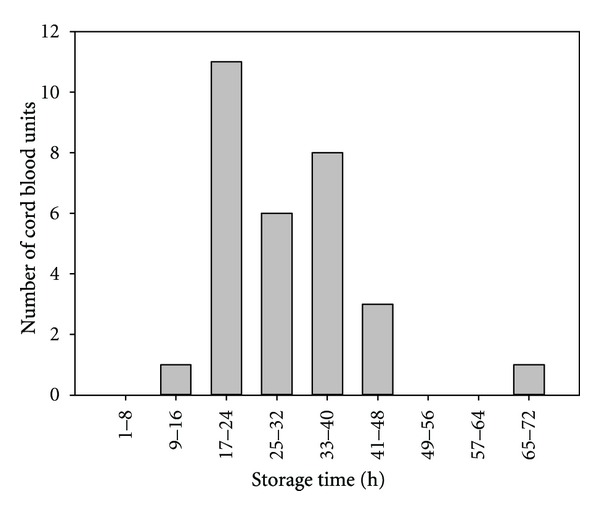
The duration cord blood samples spent at room temperature prior to testing.

**Figure 2:**
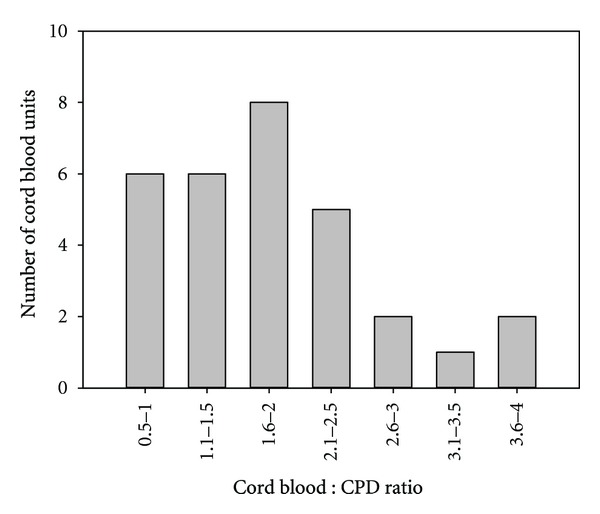
The distribution of the ratio of cord blood volume to CPD anticoagulant volume in collected cord blood samples.

A total of 30 cord RBC samples were tested in this study. Most of the samples tested were between 17 and 48 hours old (Figure 1). The average age of cord blood samples was 30.6 ± 10.3 hours (mean ± SD). The ratio of cord blood volume to the volume of CPD anticoagulant in cord blood collections ranged from 0.77 to 3.83 (Figure 2), the average ratio being 1.84 ± 0.80 (mean ± SD).

Table 1 shows correlations between cord blood characteristics and RBC quality measures. There was a fair, negative correlation between cord blood storage time and 2,3-DPG content of RBCs (*r* = −0.431, *P* = 0.017). A fair, positive correlation between cord blood hematocrit and deformability of RBCs was observed (*r* = 0.392, *P* = 0.035). Also, there was a moderately strong, positive relationship between the percentage of HbF in RBCs and their deformability (*r* = 0.668, *P* = 0.002). Other cord blood characteristics, such as baby's gender, baby's weight, cord blood volume, the ratio of cord blood volume to anticoagulant volume, and cord blood collection method, were not significantly correlated with any of the cord RBC quality measures.

Regression analysis revealed many significant effects of cord blood characteristics (predictor variables) on cord RBC quality measures (outcome variables). Regression coefficients show the nature of relationship between the predictor and the outcome. Absolute values of regression coefficients show how much the outcome changes when the predictor changes. A +/− sign before the coefficient shows the direction in which the change takes place (increase or decrease). There was a significant relationship between the age of cord blood and the 2,3-DPG concentration of RBCs, with a regression coefficient of −0.214 (*P* = 0.012). There was a significant relationship between the cord blood volume and the ATP content of RBCs, with a regression coefficient of 0.010 (*P* = 0.030). There was a significant negative relationship between the baby's weight and hemolysis, with a regression coefficient of −0.001 (*P* = 0.018). There were significant relationships between HbF content of RBCs and a number of cord RBC quality measures, such as hemolysis, with regression coefficient of −0.030 (*P* = 0.009), 2,3-DPG content, with a regression coefficient of −0.082 (*P* = 0.007), and deformability, with a regression coefficient of 0.004 (*P* = 0.002). The effect of HbF content on RBC rigidity was modified by baby's gender, so that no significant effect was observed for males; however, for females RBC rigidity decreased with the increase in HbF content (regression coefficient −0.068, *P* = 0.002). A positive relationship was observed between the ratio of cord blood volume to anticoagulant volume and PS expression on RBCs with a regression coefficient of 3.836 (*P* = 0.028). Females had an increase in CD47 expression on RBCs of 0.742% compared to males (*P* = 0.037), which was modified by cord blood hematocrit. Cord blood RBCs collected from *in utero *placenta had an increase in PS expression on their surface of 4.106% compared to RBCs collected from delivered placenta (*P* = 0.013).

A number of interactions between predictor variables were observed, wherein the effect of one predictor on cord RBC quality measure was further enhanced by a similar direction change of another predictor. Interactions between the following parameters were found: baby's weight and HbF, age of cord blood and HbF, baby's gender and HbF, the ratio of cord blood volume to the volume of anticoagulant and the method of cord blood collection, and baby's gender and cord blood hematocrit. To illustrate some of the above interactions, there was an interaction between the effect of the age of cord blood and HbF content on 2,3-DPG concentration. Since both the age of cord blood and HbF content were negatively associated with 2,3-DPG, when the age of cord blood increased, for samples with higher HbF content, the decrease in 2,3-DPG content was stronger than for samples with lower HbF content. The effect of HbF on hemolysis was modified by the change in baby's weight; in particular, cord blood samples with lower HbF content taken from smaller babies had higher hemolysis than those taken from larger babies. The effect of the ratio of cord blood volume to anticoagulant volume on RBC PS expression was modified by the method of cord blood collection, so that PS expression on RBCs collected from *in utero *placenta decreased with the increase in cord blood  : CPD ratio.

The results of comparison of cord blood characteristics and cord RBC quality measures between cord blood groups based on baby's gender and a method of cord blood collection are presented in Table 2. There were no significant differences noted in the cord blood characteristics and cord RBC quality parameters between males and females, or between cord blood collected from delivered or *in utero* placentas (Table 2).

## 4. Discussion

The goal of this study was to examine if and how the quality of RBCs isolated from whole cord blood is affected during cord blood storage at room temperature and, secondly, whether or not any of several cord blood characteristics impact the quality of cord RBCs. The primary purpose of the cord blood units used in our study was for stem cell extraction according to well-established protocols. After collection, whole cord blood is stored at room temperature for a variable amount of time dictated by the time of birth, transportation to the processing facility, and laboratory processing hours. It is then processed for stem cell isolation. Little is known about the effect of such storage conditions on the quality of RBCs present in cord blood. If cord RBCs are potentially superior for intrauterine and neonatal transfusions and are to be cryopreserved for clinical use, there is an incentive to ensure their best quality upon isolation from umbilical cord blood.

During the first 65 hours of cord blood storage at room temperature, the only observed change was a decrease in the 2,3-DPG concentration of RBCs (Table 1). Other RBC quality measures were not affected. It has been reported that 2,3-DPG disappears very quickly from adult RBCs during hypothermic storage and is usually no longer detectable by the end of the first week [55], though it is easily replenished after RBCs are transfused into the patient [56]. Therefore, if cord RBCs are to be cryopreserved for clinical use within 65 hours after cord blood collection, the drop in 2,3-DPG may not pose a serious risk for maintaining an acceptable quality of cord RBCs after cryopreservation.

Evidence in the literature suggests that the ratio of adult blood volume to the volume of anticoagulant/preservative solution is important and affects RBC quality [57, 58]. For adult whole blood collections, the standard ratio of blood volume to CPD anticoagulant volume is 7 : 1 [59]. In our study, blood : CPD ratio for cord blood units ranged from 0.77 : 1 to 3.83 : 1, due to the variable volumes obtained at the time of collection. These volumes are not predictable, and hence, an optimal cord blood  :  CPD ratio cannot be specified. A number of scientific reports indicate that the quality of adult RBCs from under- [57, 58] or over-collected blood units is suboptimal as compared with defined standard collections [58]. Evidence from our study suggests that the same may be true for cord blood collections. Particularly, lower cord blood hematocrit was correlated with lower RBC deformability (Table 1). Since cord blood hematocrit was measured after cord blood was mixed with CPD, cord blood hematocrit was partly determined by the ratio of cord blood volume to the volume of anticoagulant. Anticoagulant is acidic; therefore, when this ratio is too low (in the case of small cord blood volumes), the pH of cord blood will be reduced. This eventually results in RBC damage early in storage. A positive association between cord blood volume and ATP content, observed in the present study, also supports the argument about the importance of ratio of volumes. Higher cord blood volumes result in higher blood : CPD ratio and, therefore, higher ATP concentration. Finally, our results show that a higher baby's weight was correlated with lower RBC hemolysis. This can be explained by the fact that in our study bigger babies on average had higher cord blood volumes, which in turn resulted in higher blood : CPD ratio. On the other hand, regression analysis showed that a lower ratio of cord blood to anticoagulant was associated with lower expression of the apoptotic marker PS on RBCs. Combined, these observations lead us to believe that cord RBC quality is governed by an optimal ratio of cord blood to anticoagulant that will need to be maintained during cord blood collections for RBC transfusion *in utero* or for neonates.

We have observed the positive correlation between the content of HbF in RBCs and RBC deformability (Table 1). It is known that HbF decreases gradually during the last trimester of fetal development, and premature babies have higher percentage of HbF than full-term neonates [2]. Although the literature contains contradictory data on deformability of cord RBCs, Lindercamp et al. showed that RBCs of preterm babies have higher cellular deformability than RBCs of full-term babies [60]. Together, these two pieces of evidence are in agreement with our observation.

## 5. Conclusion

There was no significant decrease in quality measures of cord RBCs during the first 65 hours of whole cord blood storage at room temperature. The ratio of cord blood volume to anticoagulant volume in cord blood collection bag is important and needs to be optimized in order to ensure that a good quality cord RBCs are preserved. Knowledge of cord RBC quality upon isolation from cord blood is important to design procedures for cord RBC preservation. This, in turn, may result in the development of a novel blood product from a currently discarded byproduct of cord blood cell processing that may offer a superior alternative for treatment of fetal and neonatal anemias.

## Figures and Tables

**Table 1:** Correlation between cord blood characteristics and cord RBC quality measures.

Cord RBC quality measure	Cord blood characteristic							
	Age of cord blood (hours) 30.6 ± 10.3	Cord blood volume (mL) 64 ± 28	Cord blood Hct (L/L) 0.30 ± 0.04	Baby's gender	Baby's weight (g) 3468 ± 490	Cord blood collection site	HbF (%) 90.1 ± 5.8	Cord blood : CPD 1.84 ± 0.80

ATP (*μ*mol/g Hb) 2.78 ± 0.69	−0.229 (0.241)	0.337 (0.080)	0.316 (0.109)	−0.241 (0.216)	0.042 (0.841)	0.021 (0.914)	0.177 (0.497)	0.337 (0.080)
Hemolysis (%) 0.12 ± 0.04	−0.127 (0.505)	−0.144 (0.448)	−0.104 (0.590)	0.055 (0.773)	−0.085 (0.672)	−0.013 (0.946)	−0.358 (0.145)	−0.144 (0.448)
MetHb (%) 4.60 ± 1.80	0.098 (0.606)	−0.143 (0.451)	−0.236 (0.217)	0.019 (0.919)	−0.001 (0.995)	0.032 (0.866)	0.321 (0.194)	−0.143 (0.451)
2,3-DPG (mmol/L) 0.11 ± 0.20	−0.431* (0.017)	−0.133 (0.484)	−0.181 (0.346)	−0.231 (0.220)	−0.072 (0.720)	0.019 (0.919)	−0.342 (0.165)	–0.133 (0.484)
Deformability 0.446 ± 0.030	−0.119 (0.531)	0.323 (0.081)	0.392* (0.035)	0.012 (0.951)	0.254 (0.201)	0.160 (0.397)	0.668** (0.002)	0.323 (0.081)
Rigidity 1.47 ± 0.33	−0.277 (0.138)	0.010 (0.960)	−0.093 (0.630)	0.144 (0.448)	−0.127 (0.529)	0.225 (0.233)	−0.375 (0.126)	0.010 (0.960)
Microparticle events (%) 0.3 ± 0.2	−0.126 (0.540)	0.031 (0.882)	−0.240 (0.249)	−0.200 (0.327)	0.305 (0.156)	−0.088 (0.668)	0.145 (0.592)	0.031 (0.882)
PS-positive RBCs (%) 5.2 ± 0.9	0.008 (0.969)	0.015 (0.938)	−0.023 (0.910)	−0.170 (0.388)	−0.080 (0.705)	0.193 (0.325)	−0.084 (0.741)	0.015 (0.938)
CD47-positive RBCs (%) 99.9 ± 0.1	−0.144 (0.465)	−0.215 (0.271)	−0.371 (0.057)	−0.322 (0.095)	0.014 (0.947)	−0.236 (0.227)	−0.309 (0.212)	−0.215 (0.271)
PS-positive microparticles (%) 61.3 ± 18.2	−0.151 (0.461)	−0.171 (0.404)	−0.292 (0.157)	0.084 (0.682)	−0.125 (0.569)	0.104 (0.612)	−0.056 (0.836)	−0.171 (0.404)
CD47-positive microparticles (%) 88.2 ± 24.2	0.121 (0.557)	−0.076 (0.713)	−0.218 (0.295)	0.105 (0.608)	−0.209 (0.338)	0.265 (0.191)	0.137 (0.612)	−0.076 (0.713)
MFI of PS on RBCs 45 ± 10	−0.086 (0.663)	0.102 (0.606)	−0.202 (0.313)	−0.152 (0.440)	0.093 (0.660)	−0.222 (0.257)	0.344 (0.163)	0.102 (0.606)
MFI of CD47 on RBCs 274 ± 83	−0.129 (0.513)	−0.033 (0.869)	−0.105 (0.601)	−0.071 (0.718)	−0.070 (0.739)	−0.107 (0.587)	−0.070 (0.781)	−0.033 (0.869)
MFI of PS on microparticles 324 ± 55	0.135 (0.494)	0.007 (0.972)	0.155 (0.439)	0.027 (0.892)	0.035 (0.868)	−0.164 (0.403)	−0.137 (0.588)	0.007 (0.972)
MFI of CD47 on microparticles 776 ± 370	−0.168 (0.394)	−0.095 (0.632)	0.097 (0.629)	−0.080 (0.684)	−0.118 (0.574)	−0.136 (0.491)	−0.344 (0.163)	−0.095 (0.632)

The numbers in the table are correlation coefficients (*P* value in brackets), that show the strength of a relationship between two parameters. Coefficients between 0.3 and 0.5 represent a fair linear relationship, and coefficients between 0.6 and 0.8 represent moderately strong linear relationship. A +/− sign before the coefficient indicates the direction of the correlation (positive or negative). The numbers in bold are mean ± SD for each tested parameter. *Correlation is significant at 0.05 level, and **correlation is significant at 0.01 level. Hct—hematocrit, HbF—fetal hemoglobin, CPD—citrate-phosphate-dextrose (anticoagulant), ATP—adenosine triphosphate, MetHb—methemoglobin, 2,3-DPG—2,3-diphosphoglycerate, PS—phosphatidylserine, and MFI—mean fluorescence intensity.

**Table 2:** Comparison of cord blood characteristics and cord RBC quality measures between cord blood groups based on baby's gender and a method of cord blood collection.

Cord blood characteristics and cord RBC quality parameters	Baby's gender			Method of cord blood collection		
	Male (*n* = 17)	Female (*n* = 13)	*P* value	Delivered placenta (*n* = 3)	Placenta *in utero *(*n* = 27)	*P* value

Age of cord blood	30.2 (11.8)	31.2 (8.3)	0.7908	24.5 (0.4)	31.3 (10.6)	0.0028*
Cord blood volume	60 (23)	70 (34)	0.3310	61 (25)	65 (29)	0.8113
Cord blood hematocrit	0.30 (0.05)	0.31 (0.04)	0.7943	0.31 (0.03)	0.30 (0.05)	0.8676
Baby's weight	3546 (557)	3336 (333)	0.2906	3230 (407)	3498 (499)	0.3821
HbF (%)	88.5 (5.6)	91.5 (6.0)	0.2891	· (·)	90.1 (5.8)	·
Cord blood : CPD	1.71 (0.66)	2.01 (0.96)	0.3310	1.73 (0.70)	1.85 (0.82)	0.8113
ATP (*μ*mol/g Hb)	2.87 (0.58)	2.68 (0.83)	0.4811	2.68 (0.46)	2.80 (0.72)	0.7936
Hemolysis (%)	0.12 (0.05)	0.12 (0.05)	0.7987	0.11 (0.02)	0.12 (0.05)	0.6607
MetHb (%)	4.54 (1.62)	4.67 (2.07)	0.8531	4.46 (1.68)	4.61 (1.84)	0.8941
2,3-DPG (mmol/L)	0.11 (0.15)	0.11 (0.26)	0.9933	0.05 (0.03)	0.12 (0.21)	0.5923
Deformability	0.445 (0.029)	0.447 (0.032)	0.8972	0.437 (0.014)	0.447 (0.031)	0.5834
Rigidity	1.41 (0.21)	1.56 (0.43)	0.2025	1.27 (0.23)	1.49 (0.33)	0.2747
Microparticle events (%)	0.3 (0.2)	0.2 (0.1)	0.2483	0.9 (0.1)	0.3 (0.2)	0.8861
PS-positive RBCs (%)	5.4 (1.0)	5.1(0.7)	0.3842	4.7 (1.4)	5.3 (0.8)	0.2113
CD47-positive RBCs (%)	99.9 (0.1)	99.8 (0.2)	0.0706	99.9 (0.1)	99.9 (0.1)	0.4089
PS-positive microparticles (%)	61.2 (21.8)	61.5 (11.2)	0.9770	57.2 (2.4)	61.9 (19.3)	0.6848
CD47-positive microparticles (%)	87.6 (30.1)	89.2 (10.2)	0.8674	76.3 (16.6)	89.8 (24.8)	0.3736
MFI of PS on RBCs	47 (7)	44(13)	0.3924	51 (10)	45 (10)	0.2715
MFI of CD47 on RBCs	275 (73)	271 (99)	0.8983	299 (114)	271 (82)	0.5814
MFI of PS on microparticles	324 (62)	324 (46)	0.9946	366 (74)	319 (52)	0.1730
MFI of CD47 on microparticles	837 (434)	694 (258)	0.3209	899 (336)	761 (378)	0.5528

The numbers in the table are absolute values for each parameter (standard deviation in brackets), *P<0.05 (Student's *t*-test). HbF—fetal hemoglobin, CPD—citrate-phosphate-dextrose (anticoagulant), ATP—adenosine triphosphate, MetHb—methemoglobin, 2,3-DPG—2,3-diphosphoglycerate, PS—phosphatidylserine, and MFI—mean fluorescence intensity.
